# Crowdsourced Security Reconstitution for Wireless Sensor Networks: Secrecy Amplification

**DOI:** 10.3390/s19225041

**Published:** 2019-11-19

**Authors:** Radim Ostadal, Vashek Matyas, Petr Svenda, Lukas Nemec

**Affiliations:** Centre for Research on Cryptography and Security, Faculty of Informatics, Masaryk University, Brno 60200, Czech Republic; ostadal@mail.muni.cz (R.O.); svenda@fi.muni.cz (P.S.); lukas.nemec@mail.muni.cz (L.N.)

**Keywords:** ad hoc networks, crowdsourcing, cryptographic protocol, secrecy amplification (SA), wireless sensor network (WSN)

## Abstract

Research in the area of security for Wireless Sensor Networks over the past two decades has yielded many interesting findings. We focus on the topic of (re-)securing link keys between sensor nodes through so-called secrecy amplification (SA) protocols. Crowdsourcing is at the very heart of these SA protocols. Not only do SA protocols work wonders even for low-level constrained nodes with no tamper resistance, they exhibit astonishing performance in networks under significant attacker control. Our work shows that even when 50% of all network links are compromised, SA protocols can re-secure over 90% of the link keys through an intriguingly simple crowdsourcing mechanism. These protocols allow us to re-take control without any broadly coordinated cooperation, without knowledge of the compromised links, with only very limited knowledge of each particular network node and independently of decisions made by other nodes. Our article first outlines the principles of and presents existing approaches to SA, introducing most of the important related concepts, then presents novel conclusive results for a realistic attacker model parametrised by attacker behaviour and capabilities. We undertook this work using two very different simulators, and we present here the results of analyses and detailed comparisons that have not previously been available. Finally, we report the first real, non-simulated network test results for the most attractive SA protocol, our implementations of which are available as open-source code for two platforms: Arduino and TinyOS. This work demonstrates the practical usability (and the attractive performance) of SA, serving as a ripe technology enabler for (among others) networks with many potentially compromised low-level devices.

## 1. Introduction

The networking of increasingly intelligent and interconnected devices has led to the emergence of novel applications and capabilities for sensing, collecting, processing and analysing data from countless sources and environments. Wireless Sensor Networks (WSNs) embody the ultimate challenge among the various types of so-called Internet of Things (IoT) applications, as the devices (nodes) in WSNs are usually assumed to be present in large numbers, and the nodes are assumed to be as inexpensive as possible, implying limited computational and storage resources, a lack of tamper resistance to physical attacks, and limited energy sources (usually batteries). Consequently, such devices (similarly to cheap smartcards and other constrained hardware) usually have to rely on symmetric cryptography as their preferred approach (as opposed to asymmetric cryptography).

Our work targets WSNs (or indeed ad hoc networks in general) that use symmetric cryptography and link keys (keys shared between two nodes connected through a communication link; every pair of nodes shares a unique key). This is the most common setting for security in WSNs, mainly due to resource restrictions. The link keys established among neighbouring WSN nodes are an essential building block for secure communication and for more comprehensive network security applications. There are multiple ways to establish link keys, ranging from one network-wide master key, probabilistic pre-distribution [1] or plaintext key exchange [2] up to pairwise key pre-distribution. In our scenario, we assume that every pair of neighbours shares a unique (with respect to all other pairs) link key and that this key is used to encrypt all messages exchanged between the two neighbours.

Regardless of the means of establishment, link keys will always be susceptible to attackers learning them in one way or another, ranging from cryptanalytic methods up to the extraction of keys from physically captured nodes. The core purpose of our research is to improve the overall security of a network of interconnected nodes in the case that a non-trivial proportion of the link keys have become compromised (compromised key denotes a link key that has been acquired by an attacker, regardless of the point of compromise). We address this issue through so-called secrecy amplification (SA) protocols. SA protocols do not rely on any knowledge of whether a particular link has been compromised, as it is difficult, and often even impossible, to detect such a compromise given the limited resources of the nodes in a network or the nature of the attack (e.g., passive eavesdropping).

Lightweight security solutions are necessary, imposing low computational and communication overheads. To preserve protocol simplicity, we use the basic principles of crowdsourcing. Every node executes the SA protocol based only on its limited knowledge of the local network environment and layout and independently of the decisions made by other nodes. Every node is responsible for orchestrating its protocol execution with its neighbours in its close proximity and, simultaneously, for participating in the protocol execution controlled by its neighbours. Although this could appear to be a hopeless attempt without the knowledge of the compromised links and without a broadly coordinated cooperation, the results show that the nodes, as a crowd, can achieve an excellent performance. A strong majority of secure links (>90%) can be achieved using secrecy amplification protocols, even when 50% of all network links are initially compromised [3].

The core contributions of this article are as follows:

1. We verify earlier work on SA protocols (based only on simulations) through experiments in a real network.
2. We examine SA behaviour under realistic attacker models, and we determine the suitable length of amplification for the identified worst-case scenario and evaluate the corresponding resources consumption.
3. We provide tested open-source implementations of the best-performing SA protocol, HD Final, for the Arduino and TinyOS platforms.

The article is organised as follows: The second section introduces the concept of SA and also reviews related work, providing a comparative overview of various SA protocols and their properties. The third section describes a realistic attacker model that is parametrised by attacker capabilities and behaviour, concluding with an analysis of the impact of the attacker parameters on SA protocol performance. The fourth section presents the results of testbed experiments together with our implementations of the HD Final protocol for the Arduino and TinyOS platforms. Conclusions are provided in the final section.

## 2. Secrecy Amplification Principles and Related Work

### 2.1. Exploiting the Strength of the Crowd

Several research papers present the effort and results of investigating the crowdsourcing principles and their applications. The authors of [4] focused on improving the balance between signal (data) quality and crowdsourcing cost, proposing a novel incentive mechanism based on Bayesian compressive crowdsensing. A crowdsourced WiFi-based indoor positioning system was inspected in [5], including an identification of three attacks and corresponding countermeasures.

We use the crowdsourcing principles in the SA approach. Based on its limited knowledge, a particular node *A* is aware only of its own neighbours and their distances, as inferred from their signal strengths. Please note that inferring the relative distance from the received signal strength indication (RSSI) is usually a burden with errors resulting from the generally unreliable propagation of wireless signal and also as the relation between distance and RSSI is not linear. Relative distances used in group-oriented and hybrid designed protocols are robust against moderate inaccuracies as a precise node position is not required for protocols to succeed. The node can attempt to re-secure a possibly compromised link key (the link key may be compromised or not; the node cannot determine a link key’s status based on its knowledge) established with its neighbouring node *B* by trying to use a non-compromised path from node *A* to node *B*.

**Figure 1:**
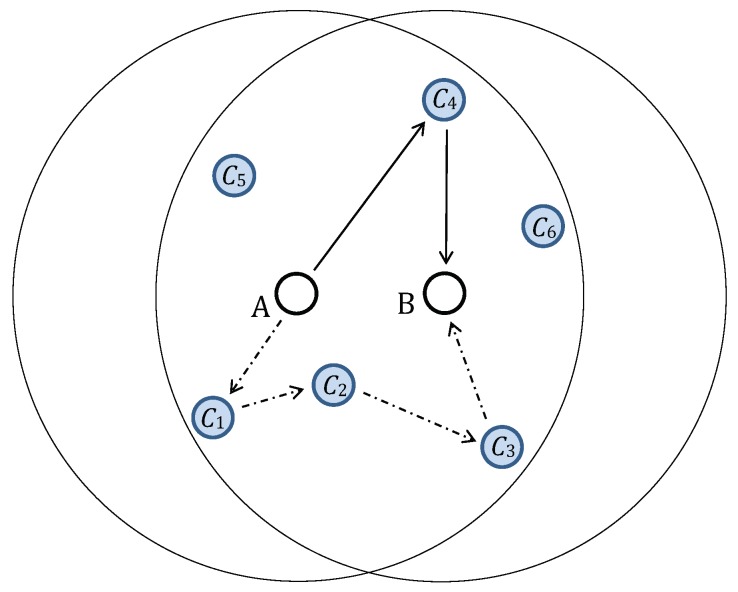
Two paths selected for a key update transmission from node *A* to node *B*. The solid lines mark a delivery path with a single selected intermediate node, $C_{4}$, and the dash-dotted lines mark a delivery path with three intermediate nodes, $C_{1}$, $C_{2}$, and $C_{3}$. The link keys used to encrypt the message are $K_{AC_{4}}$ and $K_{C_{4}B}$ in the first case and $K_{AC_{1}}$, $K_{C_{1}C_{2}}$, $K_{C_{2}C_{3}}$, and $K_{C_{3}B}$ in the second case (image from [7] © 2019 IEEE).

First, the node *A* generates a random key update with the same length as the established link key. Later on, the key update will be combined together with the current link key to create a new (secure) link key using a state-of-the-art cryptographic hash algorithm. Second, the node *A* selects one or more intermediate nodes $C_{1}$, $C_{2}$, …, $C_{N}$ (which are common neighbours of both nodes *A* and *B*) and forms the path *A* –>$C_{1}$ –>$C_{2}$ –> … –>$C_{N}$ –>*B* for key update delivery. Please refer to Figure 1 for two examples. Every node along the path would, in most cases, be a neighbour of both its predecessor and its successor. If not, the path is broken, and the key update will not be delivered. Previous research [6] has shown that even one intermediate node is sufficient to maintain the effectiveness of the protocol, using only a small fraction of the limited available resources and mostly avoiding the issue of unreachable nodes along the path. SA protocols should be robust and should yield good results even when messages are lost on delivery or when paths are broken as described above. The transmitted key update messages are encrypted using the underlying link keys in a hop-by-hop manner if such link keys already exist. Finally, nodes *A* and *B* mutually confirm that both share the same key after the exchange of key update messages.

If the key update remains unknown to an attacker, because of the attacker’s inability to either decrypt the message or correctly learn it through eavesdropping (e.g., as a result of mishearing caused by a packet collision), then the link key between nodes *A* and *B* is updated and re-secured against the attacker.

A secrecy amplification protocol specifies a procedure for selecting a particular neighbour for a key update delivery and determining a sequence of intermediate nodes to form a delivery path and governs the number of protocol repetitions and the length of execution. The resulting SA protocol is a trade-off between its resource requirements (e.g., the energy required for message transmission) and its ability to improve the number of secure links in the network. The overall design goal is to develop SA protocols that can secure a high number of links yet require only a small number of messages to save precious energy during radio transmissions. Thus, such a protocol needs to be as simple as possible.

From the attacker’s point of view, she initially has access to several compromised link keys (by means of cryptanalytic methods, the extraction of keys from physically captured nodes, eavesdropping on initial key establishment, etc.). To maintain such a compromise, she needs to eavesdrop on as many secrecy amplification messages as possible. This requires constant monitoring of the entire network, as the amplification process can be executed multiple times during the network’s lifetime. If the attacker loses track of one link key that is re-secured, then it can and will be used to transmit more key updates to its neighbours later in the execution of the protocol. The more non-compromised links exist in the network, the faster the convergence towards a secure network will be.

SA protocols have also recently been exploited in a combination with key extraction from radio channel fading, with promising results for certain types of networks and their operational environments [7,8].

Different classes of SA protocols use different capabilities to improve security throughout a network. Although all SA protocols attempt to establish new (possibly more secure) link keys, three main distinct classes of SA protocols exist.

### 2.2. Node-Oriented Protocols

Node-oriented protocols were first introduced by Anderson et al. in [2] to provide an additional layer of protection after plaintext key exchange in a key distribution approach called *key infection*. Such a protocol is very simple; it sends key updates via every possible neighbour. The main advantages of node-oriented protocols are the simple synchronisation of multiple protocol executions running in parallel and their generally low memory overheads.

A node-oriented protocol is executed for all possible *k*-tuples of neighbours in the network, and the number of such *k*-tuples can be high, especially in a dense network. This is the most limiting property of node-oriented protocols, as it results in an enormous number of messages being sent by every node. The number of messages increases polynomially with respect to the number of neighbouring nodes and exponentially with respect to the number of parties participating in the protocol (the number of intermediate nodes along every path from node *A* to node *B*).

The first node-oriented protocol, denoted as the Push protocol, was presented by Anderson et al. in [2]. Liu et al. used the Push protocol as a basis for an establishment of the intra-group link keys between multiple nodes belonging to different groups, where a more structured deployment was assumed [9]. A variant of the initial key exchange mixed with the Push protocol (denoted as Commodity) without an explicit SA was presented by Kim et al. in [10], together with a formal security proof. The fraction of secured links was lower than for the Push protocol alone. A multi-hop version of the Push protocol was analyzed by Liu et al. in [11]. Variants of the Push and Multi-hop Push protocols called Pull and Multi-hop Pull protocols were presented by Cvrcek et al. in [12]. The best-performing node-oriented protocol (NO Best) was presented by Svenda et al. in [3] and more information about that protocol is provided in the Appendix A.1.

### 2.3. Group-Oriented Protocols

Group-oriented protocols were first proposed by Svenda et al. in [3] to decrease the number of messages sent during an amplification protocol to overcome the main limitation of node-oriented protocols. Neighbouring nodes share key update values within a larger group of cooperating nodes identified by their geographic locations with respect to nodes *A* and *B* (to re-secure the key $K_{AB}$). In a previous study, group-oriented protocols were automatically generated using linear genetic programming [13], and the SensorSim network simulator developed by the authors of [3] was then used to evaluate the quality of the candidate protocols.

Group-oriented protocols require a higher level of cooperation and information sharing among neighbouring nodes. The crucial disadvantages of group-oriented protocols are the challenge of synchronising parallel executions and the complexity of the security analysis due to the high number of nodes involved. Consequently, neither a detailed evaluation nor even an implementation (outside of simulators) of group-oriented protocols has ever been reported.

The best-performing group-oriented protocol (GO Best) was presented by Smolka et al. in [14] and more information about the protocol is provided in the Appendix A.2.

### 2.4. Hybrid Designed Protocols

Hybrid designed protocols [6] combine the advantages of both node- and group-oriented protocols. They are constructed using genetic programming in combination with manual post-processing. They take advantage of knowledge obtained through both node- and group-oriented protocols (hence the term hybrid design) and statistical data about the most suitable placement of the participating intermediate nodes. Hybrid designed protocols use both sub-protocols (similarly to node-oriented protocols) and relative distances (similarly to group-oriented protocols) and perform several repetitions of the entire process to achieve the required success rate.

The resulting protocols are very simple and require less cooperation compared with group-oriented protocols. They outperform both node- and group-oriented protocols with respect to their success rates while sending fewer messages. They are easy to analyse and implement and enable simple synchronisation and parallel execution.

Examples of hybrid designed protocols include HD Final and HD Best [6]. More information about both protocols is provided in Appendix A.3 and the implementation of the HD Final protocol is discussed later in Section 4.2.

### 2.5. Protocol Comparison

**Figure 2:**
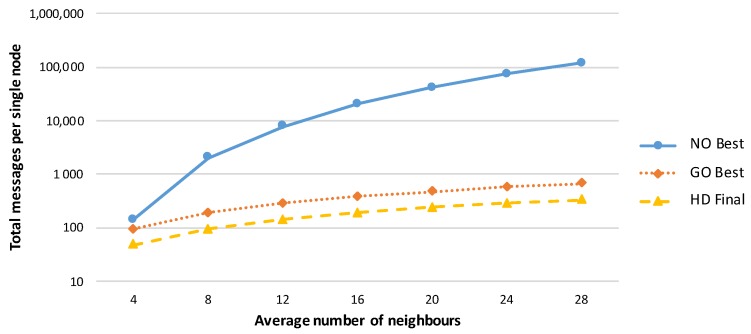
Total numbers of messages required per node in the best node-oriented, best group-oriented, and final hybrid designed secrecy amplification protocols (under the assumption of a network with 7.5 neighbours per node on average). The group-oriented and hybrid designed protocols send considerably fewer messages compared with the node-oriented protocol.

**Figure 3:**
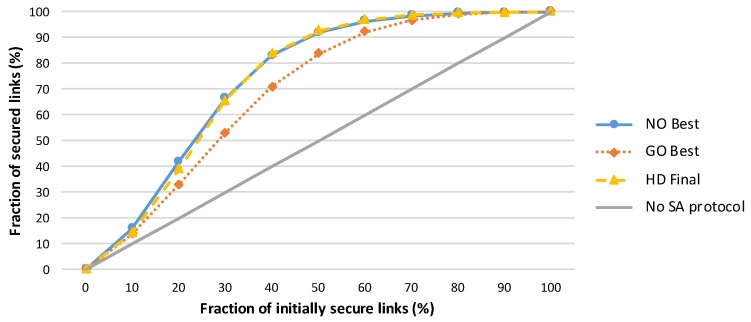
Increases in the number of secured links after the secrecy amplification protocols are executed. A strong majority of secure links (>90%) can be achieved using a secrecy amplification protocol, even when 50% of all network links are initially compromised. The performance of the node-oriented and hybrid designed protocols are comparable, and both significantly outperform the group-oriented protocol. This graph represents an average of 50 simulations of network run, where the network consists of 1000 randomly distributed sensor nodes (7.5 neighbours per node on average). A simple attacker model (as described in Section 3.2) with the random compromise pattern is assumed.

**Figure 4:**
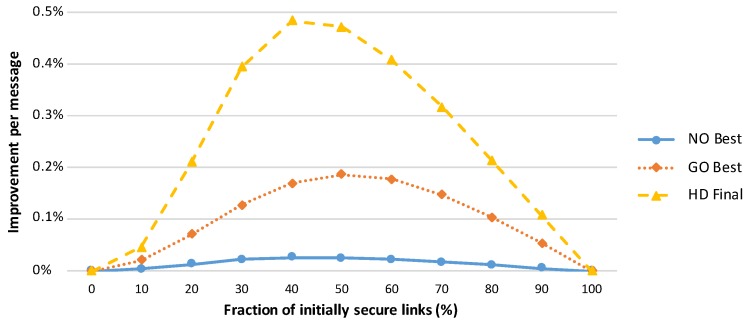
Increases in the ratio of secured links per message exchanged during protocol execution. The node-oriented protocol sends significantly more messages, and consequently, this protocol is the least efficient. The best trade-off is observed for the hybrid designed protocol. This graph represents an average of 50 simulations of network run, where the network consists of 1000 randomly distributed sensor nodes (7.5 neighbours per node on average). A simple attacker model (as described in Section 3.2) with the random compromise pattern is assumed.

A comparison of various amplification protocols with respect to the number of messages sent is presented in Figure 2. The success rates of the amplification protocols executed in networks with different numbers of initially compromised links are shown in Figure 3, and the effectiveness of the protocols is compared in terms of the security gained per message sent during amplification protocol execution in Figure 4.

A detailed comparison of the properties of different amplification protocols and their results for multiple compromise patterns and for networks with different densities are provided in a paper by Ostadal et al. [6].

We used the simplified simulator SensorSim [3] of Svenda et al. during the initial phase of our SA protocol research. This simulator enabled the use of genetic programming during the protocol proposal stage and permitted an extensive evaluation of the average protocol performance for different network layouts and in different scenarios. The main advantage of SensorSim is the speed of simulation. However, this simulator lacks many essential components for realistic network simulations, such as radio signal propagation and MAC layer collisions.

## 3. Attacker Behaviour and Capabilities

During the initial research of SA protocols and the identification of the major node-oriented, group-oriented and hybrid designed protocols of interest, a weakened attacker model and subsequent simple attacker model were used. Both came with very simplified and unrealistic properties allowing only basic SA protocol examination and evaluation. Later, a further development of a realistic attacker model was necessary for the evaluation and verification of the SA protocols’ properties and performance. We formulate a set of realistic attacker characteristics in terms of attacker behaviour and capabilities, and we evaluate the performance of several major SA protocols based on this advanced, realistic attacker model. Please note that we exclude group-oriented protocols from the subsequent comparisons because of the issues described in Section 2.3 (e.g., complex synchronisation and implementation).

Together with the new attacker model, a more advanced and realistic network simulator was needed. We extend the KMSforWSN framework, a tool for automated evaluation of the properties of key management schemes (KMSs) in WSNs, which is built on top of MiXiM [15], a WSN framework for the OMNeT++ simulator [16]. This framework was introduced by Jurnecka et al. in [17], and our extension is available as an open-source tool (http://crcs.cz/papers/cans2016).

### 3.1. Weakened Attacker Model

For the following discussion, we need to explain the *weakened attacker* model. The weakened attacker model assumes that the attacker is able to monitor only a fraction of links for a short interval. This assumption is valid only for a certain period of time after deployment and then we have to consider a stronger attacker with the ability to eavesdrop all communication. The attacker with a limited number of eavesdropping devices can eavesdrop only a fraction of links and the rational reason behind this assumption is based on specifics of WSNs:

- Locality of eavesdropping: The low communication range of nodes allows for a frequent channel reuse within the network and detection of extremely strong signals, so it is not possible for an attacker to place only one eavesdropping device with a highly sensitive and strong antenna.
- Low attacker presence during deployment: A low threat in most scenarios during the first few seconds before the attacker realizes what target area is in use. If the attacker nodes are already present in a given amount in the target location, we can deploy a network with density and node range such that the ratio between legal nodes and the attacker’s eavesdropping devices is such that a secure network can be formed.

### 3.2. Simple Attacker Model

A simple attacker model considers compromised networks that exhibit only two different compromise patterns: random compromise and key infection.

A random compromise pattern is the result of a node-compromise attacker model together with a probabilistic pre-distribution key establishment scheme [1]. In this model, the attacker captures a fraction of the deployed nodes and extracts keying material from captured nodes. Because of the probabilistic pre-distribution mechanism, the attacker may be able to compromise additional links based on the extracted keying material.

The key infection pattern assumes a weakened attacker model [2] together with a key establishment mechanism in which link keys are exchanged in plaintext.

After the initial compromise, we assume a global passive attacker who is able to monitor all communications in the entire network.

### 3.3. Realistic Attacker Model

A realistic attacker is parametrised by her capabilities and behaviour [18]. She does not have global coverage of the network, but she is able to eavesdrop on messages based on her equipment and current position. The attacker parameters can be divided into two separate groups: behaviour parameters and resource parameters.

The behaviour parameters characterise the attacker’s strategy and behaviour during her activity. We investigated various initial compromise patterns (established by the attacker extracting keying material from selected nodes), ranging from the compromise of random nodes up to a case in which the attacker traverses the network and selects nodes along her trajectory (e.g., nodes around the border or at the centre of the network or nodes along a path from the border to the centre). The movement pattern of the attacker describes the attacker’s eavesdropping activity during the execution of the SA protocol. The investigated patterns range from a random walk and linear and circular patterns up to the coordinated patrolling of a targeted area. Multiple initial attacker locations and different attacker movement speeds were investigated.

The resource parameters define the available resources and capabilities of the attacker. We investigated the SA protocol performance for cases in which several attackers work together to eavesdrop on as many communications as possible. The eavesdropping range depends on the available equipment and its sensitivity, and it strongly influences the attacker’s success. The last investigated parameter corresponds to the attacker’s ability to infect a WSN node with malware when initially compromising the node. Such a node is then under the attacker’s control, but the control remains passive, aside from providing a monitoring functionality, the malware does not affect any behaviour of the node. We investigated the impact of an increasing number of infected nodes in the performance of the SA protocols.

### 3.4. Impact of Attacker Models on the SA Protocols

It was necessary for us to overcome the limitations imposed by the simplified simulator prior to further evaluation. We used an extended version of the KMSforWSN framework together with definitions of the channel and physical layer settings based on previous research of Stetsko et al. on the real parameters of TelosB sensors for an outdoor environment [19]. We simulated the network execution not only as a graph discovery problem (as in SensorSim), but also through a full emulation of the code running on virtual nodes, with execution of the application logic and the passing of messages to the communication stack. The simulation encompassed realistic attacker behaviour and capabilities, including movement patterns and equipment sensitivity.

Ostadal et al. performed an extensive number of realistic simulations of various scenarios and attacker models. They determined a ranking of the major amplification protocols of interest based on their performance in a prevalent number of investigated cases. The hybrid designed protocols outperformed the rest in all scenarios we examined, and these protocols were found to be robust across different attacker behaviour patterns and capabilities. Please note that the NO Best protocol produces almost the same results as the HD Final protocol, but at the price of an enormous increase in the number of messages sent.

**Figure 5:**
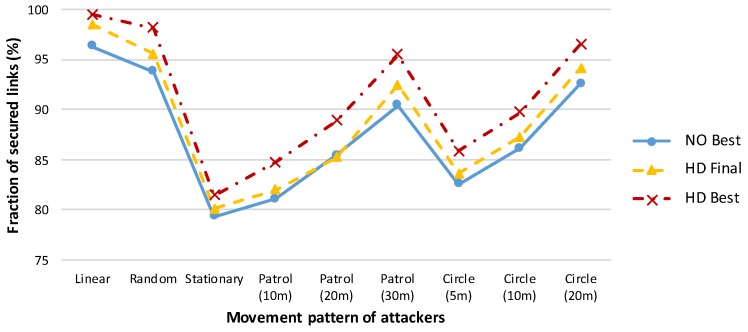
Success rates of amplification protocols for various attacker movement patterns. The numbers shown in brackets for the *Patrol* and *Circle* patterns denote the side length of the square patrolled area and the diameter of the circle, respectively. The initial compromise rate is 50% of all link keys [18].

Simulator-based results of Ostadal et al. indicate that *the most favourable strategy for an attacker is to remain in one place* throughout the entire secrecy amplification process, as she is able to eavesdrop on all communications within a particular area. Any movement leads to a reduction in the set of compromised keys in any area from which she leaves because of missed transmissions of SA protocol messages with fresh keys. Figure 5 presents the results of the investigated SA protocols for various attacker movement patterns. Those findings served as the basis for our implementation of the hybrid designed protocol HD Final on several platforms to facilitate its broader usage and to enable our testbed experiments.

A detailed evaluation of Ostadal et al. of attacker behaviour and capabilities is presented in [18].

## 4. Testbed Experiments and Results

Even the most detailed simulator can only approximate a real environment, most notably because of the incomplete specification of complex radio propagation behaviours and the various limitations of real-world devices, including the behaviour of software stacks.

To validate the simulated results, we performed several experiments using real WSN nodes in a testbed network with three main goals. Our purpose was to verify the results, performance, and expected benefits of the SA protocols in a real environment. Furthermore, we wanted to investigate the impact of the attacker strategies, behaviour patterns and capabilities described at the end of Section 3.

We also wanted to investigate protocol performance and to determine a suitable duration (together with a suitable number of protocol repetitions) of the HD Final protocol to achieve reasonable network security (more than 85% secured links) in the worst-case scenario. The worst-case scenario corresponds to the initial compromise of all link keys and the most favourable attacker strategy, in which she starts at a position from which she can monitor the entire network and remains stationary.

Last but not least, we provide implementations of the HD Final protocol for two selected platforms, Arduino [20] and TinyOS [21], which are available online as open-source implementations. We selected the HD Final protocol because of its superiority compared with the node- and group-oriented protocols, based on the comparisons presented in Section 2. Compared with the HD Best protocol, HD Final sends significantly fewer messages, yet achieves a sufficiently high success rate.

### 4.1. Testbed Network and Experimental Setting

Our testbed network consisted of 24 legitimate nodes placed below the ceiling in six adjacent rooms. We performed the experiments using JeeLink Classic v3 devices (http://jeelabs.net/projects/hardware/wiki/JeeLink). Every node was equipped with an ATmega328p AVR microprocessor (Atmel) and an RFM12B wireless radio module (HopeRF), operating at a frequency of 868 MHz. The topology of the network was known in advance; each node had 7.83 neighbours on average, resulting in a total of 94 links in the network.

The reference length is a necessary parameter for the execution of a hybrid designed protocol (used for the selection of intermediate nodes); it represents the approximate distance between the most distant pair of neighbours in the network. We experimentally determined this length to be 13 meters. This value will be different for every network, as it is highly dependent on the environment, the placement of the WSN nodes and the hardware used. The duration of the amplification protocol was set to five minutes to achieve a reasonable trade-off between the execution time being too short (increasing message collisions) or too long (limiting normal network use).

**Figure 6:**
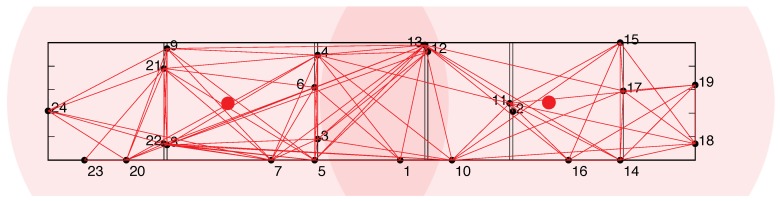
Our network layout, consisting of 24 legitimate nodes, indicated by black points and their corresponding indexes. The nodes are placed below the ceiling in six adjacent rooms. The initial positions of the attackers are marked with red points. These positions enable the attackers to monitor all legitimate communications between every pair of neighbours.

We investigated a scenario with two cooperating attackers, each covering approximately half of the network. Both attackers were equipped with hardware equivalent to that used by legitimate nodes. The network layout and attacker starting positions are shown in Figure 6. All presented results are averages over 10 repeated measurements in our testbed network.

### 4.2. Arduino and TinyOS Implementations

We provide implementations of the HD Final protocol for two widely used platforms: Arduino and TinyOS. Both implementations have been released as open-source code in a Git repository, available through this article webpage (https://crocs.fi.muni.cz/papers/iot2018).

The protocol consists of two phases, and the user is expected to define three basic configuration parameters: an amplification length, a maximum number of neighbours, and the reference length (described above). The amplification length parameter has a significant impact on the amount of message collisions and a largely reduced amplification length could result in HD Final protocol failure. The maximum number of neighbours determines the size of data memory required to store a neighbour table. The node maintains the following information for every neighbour: neighbour ID [8 bits], first intermediate node ID [8 bits], second intermediate node ID [8 bits], shared key [128 bits], key update generated [128 bits], and key update received [128 bits]. A large amount of neighbours could deplete the memory of a low end devices, such as TelosB.

In the first phase, the HD Final protocol requires information about the neighbours of the node on which it is running and their respective distances. The coarse distances may be obtained through received signal strength indication (RSSI) measurements, or the network topology may be known in advance. Our implementation is currently designed for a known network topology, but it could be easily updated to consider RSSI measurements (where such measurements are possible for the given hardware). The node itself (master) identifies two intermediate nodes for every neighbour (slave) based on the measured distances. The process requires knowledge of all mutual neighbours of master and slave nodes and the measured distances of every intermediate node from both master and slave. In a case that no mutual neighbour exists, the direct link between master and slave is used. The total number of key update messages is calculated based on number of neighbours, and the dispatching of messages is planned uniformly over the defined protocol duration.

In the second phase, the master node generates key update messages and sends them according to the timing calculated in the first phase. Every key update value is at the same time stored in the neighbouring table in the key update generated field for respective node. The key update will be used later to update the shared key when confirmed by the slave node. A key update message consists of the key update value, the ID of the master node, the ID of the intermediate node (which only forwards the message) and the ID of the slave node. Every subsequent message is sent to a different neighbour, using a round robin approach on the neighbour table. This approach provides a sufficient time for message processing by the slave node and for the key update confirmation. Once the message is received by the slave node, the node generates and sends a nonce confirmation message consisting of the master ID and slave ID. The master updates the mutual shared key when the nonce confirmation message is received. The slave updates the mutual shared key when the acknowledgement for nonce confirmation message is received. The HD Final protocol finishes when all key update messages were sent and processed.

Both implementations provide simple and reliable message delivery with up to four message retransmissions in the case that a message is lost. All messages sent during protocol execution are acknowledged. All messages and retransmissions are sent with a small random delay to limit the amount of message collisions.

### 4.3. Practical Examination of Published Results

The goal of the first experiment was the verification of the performance of the HD Final protocol and of the most favourable attacker strategy for maintaining a compromised state in a WSN network. We investigated three different attacker settings: (1) The attacker remains stationary in her starting position and does not move at all. (2) The attacker patrols a small area around her starting position (in a range of approximately 1.5 metres), remaining in the same room. (3) The attacker patrols a larger area including locations more distant from her initial starting position, even visiting the two adjacent rooms. Moreover, we investigated the performance of the HD Final protocol for an attacker starting outside the rooms and patrolling the corridor along the bottom side of Figure 6.

We considered two different compromise patterns. (1) Random nodes are compromised, with up to 50% of links being compromised. When a node is compromised, all keying material is extracted, but the node continues to perform legitimately in all subsequent operations. Using this process, 53% of the link keys were compromised on average during our experiment. (2) The worst-case scenario, in which the keys from all nodes are compromised, resulting in 100% of the links being initially compromised.

**Figure 7:**
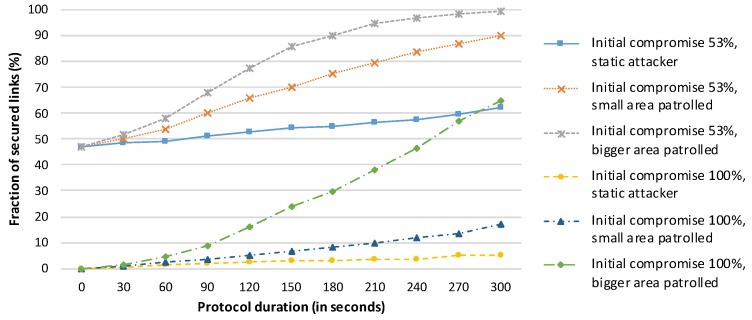
Performance of the HD Final protocol for an attacker starting in a suitable position within the network, as shown in Figure 6.

The experimental results are shown in Figure 7 for an attacker starting within the WSN. We can observe a security improvement provided by the SA protocol on the real hardware and in the real environment. There is a proven benefit to using SA protocols, HD Final in particular. Execution of the HD Final protocol improves network security, but the success rate strongly depends on the attacker’s behaviour. We observe only a small improvement for the case in which all links are compromised (approximately 3.5% improvement), but a larger improvement for the case in which 53% of the links are compromised (approximately 15% improvement). These findings illustrate the strength of SA protocols using non-compromised paths.

**Figure 8:**
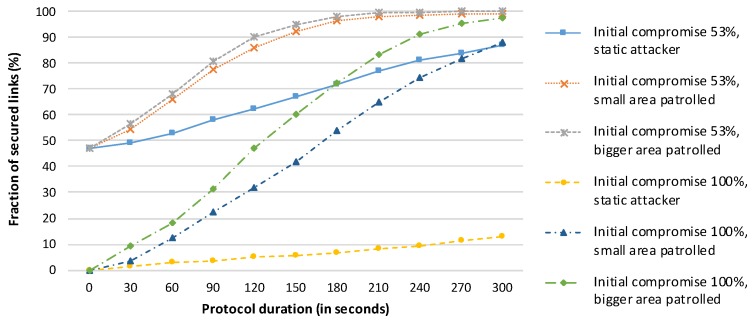
Performance of the HD Final protocol for an attacker starting outside the network in the corridor running along the bottom side of Figure 6.

The experimental results for an attacker starting outside the network in the corridor are shown in Figure 8. The success rates of the HD Final protocol are greatly improved as a result of the disadvantageous position of the attacker. The success rate is greater than 85% even for the case in which 100% of the link keys are initially compromised, provided that the attacker does not remain stationary.

These results provide clear confirmation that the most beneficial strategy for the attacker is to remain stationary and consistently eavesdrop a particular area. Any kind of attacker movement results in a non-monitored part of the network (the area being eavesdropped by the attacker positioned before the movement and not being monitored after the movement) that is immediately re-secured by the SA protocol. Even a short period of time (before the attacker returns to the original position) is sufficient for a significant improvement of the success rate.

This verifies the findings from [18] that were based on simulations. Furthermore, the larger is the area patrolled by the attacker, the fewer links remain compromised and, consequently, the higher is the SA success rate. This confirmation is consistent with the results of Ostadal et al. presented in Figure 5.

The small improvement achieved in the worst-case scenario, in which all link keys are compromised and the attacker remains stationary at the most suitable position, is due to packet collisions and interference causing the attacker to mishear eavesdropped communications. This was the main motivation for the experiment reported in the following section.

### 4.4. SA Protocol Performance in the Worst-Case Scenario

**Figure 9:**
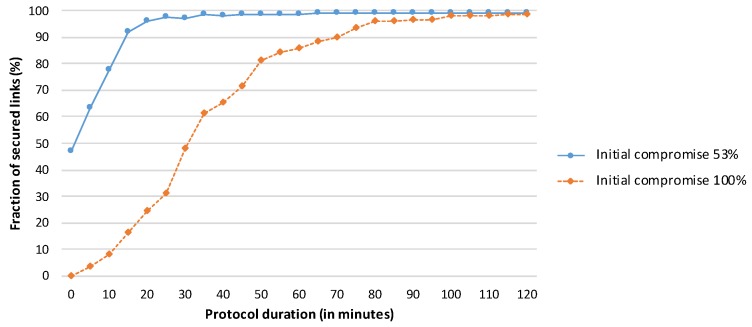
Performance of the HD Final protocol when the attacker starts in a suitable position within the network, as shown in Figure 6.

The objective of this experiment was to identify the number of repetitions of the HD Final protocol (and the protocol duration) required to achieve reasonable network security (more than 85% secured links) for the scenario in which *all links* are initially compromised. We repeated the complete HD Final protocol 24 times, with the resulting execution time of 2 h. We also include an evaluation of the protocol performance for the case where 53% of the links (instead of all links) are initially compromised as a reference for comparison. The results are presented in Figure 9.

The HD Final protocol results in a reasonably secure network after 15 min for the case in which half of the links are initially compromised and after one hour for the worst-case scenario. Moreover, the protocol is able to ensure that 98% of the links are secured after 100 min in the worst-case scenario. The average numbers of messages sent per node are 282 for a protocol duration of 15 min, 1128 for a one-hour run, and 1880 for a 100-minute execution time.

The increase in the number of secured links is initially rapid, as the protocol can immediately use the newly secured links to its advantage. As the network approaches the state in which most links are secured, the increase slows, as not all secured paths are used (the HD Final protocol identifies only two intermediate nodes) and it may be more difficult to secure particular links (e.g., because of a lack of common neighbours for a neighbouring pair on the border of the network).

## 5. Conclusions

Our work reviews the evolution of SA protocols, from their first conception up to the real implementation of the best-performing protocol, HD Final, for the Arduino and TinyOS platforms to enable facile integration into real-world applications. Using the principles of crowdsourcing, the HD Final protocol is executed independently of the decisions made by other nodes and even without knowledge of whether a particular link is compromised, resulting in a very simple protocol that permits easy synchronisation and parallel execution.

The attacker model was investigated in detail, and realistic attacker capabilities and behaviour were considered, concluding with an identification of the worst-case scenario. The HD Final protocol was shown to be robust against different attacker parameters, providing great benefit even in the worst-case scenario. The HD Final protocol can ensure that 98% of all links are secured after 100 min (with 1880 messages sent per node on average) when it is executed in a network in which all link keys are initially compromised and with the attacker covering the entire network and remaining stationary. We conducted testbed experiments in a real WSN, obtaining findings that confirmed the simulation results.

One of the key characteristics of SA protocols is their good performance even when a non-trivial proportion of the link keys are compromised. SA protocols can be used either in reaction to a network compromise or even as a preventive measure as part of an overall layered security strategy. Another usage mode is to execute an SA protocol after plaintext key exchange in a newly established network, thereby ensuring the required level of network security with a reasonable investment of time and energy resources.

Considering the simplicity of SA protocols and the benefits they provide, we expect that their use cases should not be limited to the IoT world. As crowdsourced security has and will have many other applications, our findings and implementations may also find use in different applications based on or related to key re-securing/updating.

## Abbreviations

The following abbreviations are used in this manuscript:

SA	Secrecy amplification
WSN	Wireless sensor network
IoT	Internet of Things
KMS	Key management scheme
RSSI	Received signal strength indication

## Appendix A. Detailed Description of Discussed Protocols

We provide a detailed description and a pseudo-code of discussed SA protocols: NO Best, GO Best, HD Best and HD Final.

Every network node orchestrates the protocol execution in the role of a *master* node. The *master* node continuously selects *slave* nodes from its neighbours. The link key shared between the *master* and *slave* nodes is updated. Several other neighbouring nodes could participate in the protocol in the role of *intermediate* nodes. *Intermediate* nodes do not update any link keys in node-oriented and hybrid designed protocols. Link keys among all participating nodes are updated in case of group-oriented protocols.

Every node in the protocol is modelled as a computing unit with a limited number of memory slots. Each memory slot can contain either a key update generated on the node or the key update received from a neighbouring node. SA protocol is then a sequential chain of primitive instructions, generating key updates into memory slots and exchanging key updates among nodes. The two instructions being used follow:

- RNG$N_{a}$$R_{i}$ – generate a random key update on node $N_{a}$ into slot $R_{i}$.
- SND$N_{a}$$N_{b}$$R_{i}$$R_{j}$ – send a value from $R_{i}$ on node $N_{a}$ to slot $R_{j}$ on $N_{b}$.

### Appendix A.1. NO Best Protocol

The NO Best protocol is 4-party node-oriented protocol, requiring the involvement of the *master* node, the *slave* node, and two *intermediate* nodes. The pseudocode of the NO Best protocol is provided in Protocol 1. We assume that every node is aware of its neighbours before the protocol is executed.

Every node executes the NO Best protocol in a role of the *master* node (row 1). The *master* node selects iteratively *slave* nodes (row 3) and two *intermediate* nodes (rows 4 and 5) from its neighbours. After the determination of all parties, the *master* node informs the *intermediate* node 1 about its role (row 6) as the node is responsible for key update generation.

Three key updates are generated during the execution of the NO Best protocol for every selected 4-tuple: two by the *master* node and one by the *intermediate* node 1. Key updates need to be shared by both *master* and *slave* nodes, as the two nodes will later update the shared link key. The first key update is generated by the *master* node and sent directly to the *slave* node (rows 7 and 8). The second key update is generated by the *master* node and sent to the *intermediate* node 2; the *intermediate* node 2 delivers the key update to the *slave* node (rows 9–11). The third key update is generated by the *intermediate* node 1. The key update is delivered directly to the *master* node and to the *intermediate* node 2. *Intermediate* node 2 finally sends the key update to the *slave* node (rows 12–15). All three key updates are independent and the order of the delivery is not important.

*Master* and *slave* nodes mutually confirm every received key update. The key updates are used to update the link key after the successful confirmation (row 16).

**Protocol 1** Pseudocode of the NO Best protocol.

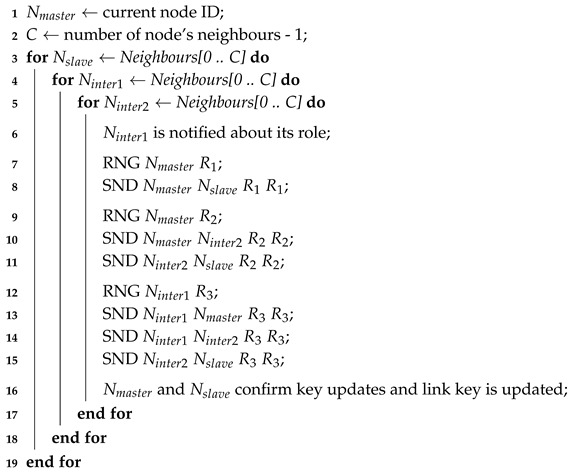

### Appendix A.2. GO Best Protocol

The GO Best protocol is a group-oriented protocol requiring the involvement of the *master* node, the *slave* node, and up to 33 *intermediate* nodes. The number of intermediate nodes is highly dependent on the number of *master* node’s neighbours. The pseudocode of the GO Best protocol is provided in Protocol 2. We assume that every node is aware of its neighbours and of the distances between the node and every neighbour before the protocol is executed. Those distances can be obtained through received signal strength indication (RSSI) measurements, or the network topology may be known in advance.

Every node executes the GO Best protocol in a role of *master* node (row 1). The whole group of neighbours (the *master* node and all its neighbours) allocate 12 memory registers for the amplification run with this particular *master* node (row 3). The memory slots of the neighbours involved (for the same *master* node) are not cleared between the protocol executions with different *slave* nodes. This enables the group-oriented protocol to propagate key updates among a group of neighbours. This requirement results in total 12∗(numberofneighbours+1) memory registers to be allocated by every node in the network.

The *master* node selects iteratively the *slave* nodes from its neighbours (row 4). The *slave* node provides a list of distances from all its neighbours to the *master* node (row 5). Based on the actual deployment of nodes, parties of the protocol are replaced by real identification of the nodes that are positioned as close as possible to the relative identification given by *master* and *slave* nodes in the protocol (row 6). All participating nodes are informed by the *master* node about their roles in the protocol (row 7). Please note that one real node can be assigned multiple roles in case this node is the nearest one with respect to multiple relative distances. Multiple key updates are generated and shared within the group of participating nodes (rows 8-45). Finally, the link keys are updated among every pair of neighbours within the group of nodes participating in the protocol for particular *master* and *slave* nodes (row 46). Please note that the memory registers are not erased and all stored values could be used in the iteration with the next *slave* node.

**Protocol 2** Pseudocode of the GO Best protocol.

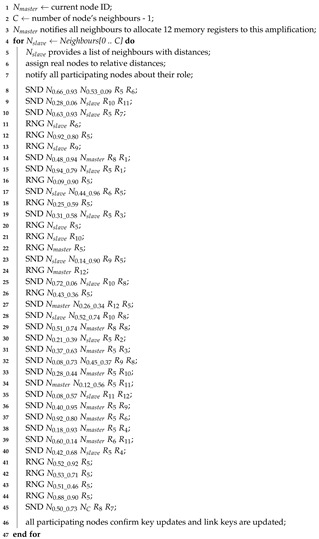

### Appendix A.3. HD Final and HD Best Protocols

HD Final and HD Best are hybrid designed protocols requiring the involvement of the *master* node, the *slave* node, and *intermediate* nodes, 2 and 5 respectively. The number of intermediate nodes is the only difference between the two protocols. Removing 3 intermediate nodes from the HD Best protocol saves 6 messages in every protocol iteration for only a negligible drop of the success rate. The pseudocode of HD Final and HD Best protocols is provided in Protocols 3 and 4, respectively. We use the HD Best protocol as the reference protocol in the following paragraphs. Nonetheless, all statements hold also for the HD Final protocol. We assume that every node is aware of its neighbours and of the distances between the node and every neighbour before the protocol is executed. This is the same assumption as in the case of group-oriented protocols.

Every node executes the HD Best protocol in a role of the *master* node (row 1). The whole protocol is repeated three times (row 3). The *master* node selects iteratively *slave* nodes (row 4). Each *slave* node provides a list of distances from all its neighbours to the *master* node (row 5). Based on the actual deployment of nodes, parties of the protocol are replaced by real identification of the nodes that are positioned as close as possible to the relative identification given by *master* and *slave* nodes in the protocol (row 6). There is no requirement to inform *intermediate* nodes about their roles, as all the nodes only retransmit messages towards their destination.

Five key updates are generated for each combination of *master* and *slave* nodes. Every key update is generated by the *master* node and shared with the *slave* node through a different *intermediate* node. A detailed description of the process together with relative distances of *intermediation* nodes is provided in pseudocode rows 7–21. All five key updates are independent and the order of the delivery is not important. *Master* and *slave* nodes mutually confirm every received key update. The key updates are used to update the link key after the successful confirmation (row 22).

**Protocol 3** Pseudocode of the HD Final protocol.

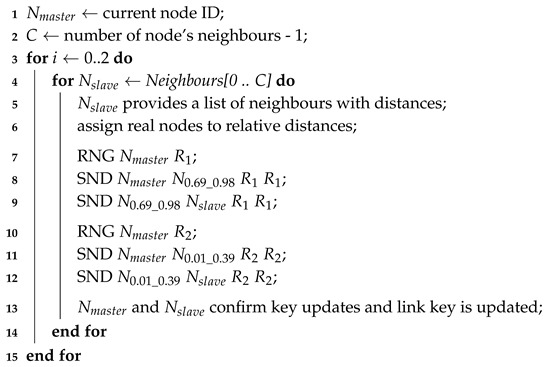

**Protocol 4** Pseudocode of the HD Best protocol.
